# Patient views on the effectiveness of audio-dentistry for emergency triage during COVID-19

**DOI:** 10.5339/qmj.2025.13

**Published:** 2025-02-27

**Authors:** Shaymaa Abdulreda Ali, Sahar Alaji, Ayah Abdallah Alqaisi, Reem Binrabbaa, Moudi Dakhel Alkhaldi, Suhayla Reda Al Banai, Najah Alhashimi

**Affiliations:** Hamad Medical Corporation, Doha, Qatar *Correspondence: Shaymaa Abdulreda Ali. Email: shaymaabdulreda@gmail.com

**Keywords:** Audio-dentistry, teledentistry, teletriage, dental emergencies, patient satisfaction, COVID-19

## Abstract

**Background:**

When Qatar imposed a nationwide lockdown in accordance with WHO guidelines during the first wave of the COVID-19 pandemic, dental healthcare services were disrupted, limiting services to emergencies and postponing elective procedures due to transmission risks. Teledentistry was introduced to remotely manage dental conditions and reduce hospital admissions. The present study examines patient perceptions of audio-dentistry, a form of teledentistry, in managing dental emergencies during the pandemic and explores factors influencing overall patient satisfaction.

**Methods:**

A retrospective, cross-sectional telephone questionnaire included 352 participants who used a dental emergency hotline service during the first wave of the COVID-19 pandemic lockdown (March 29–August 31, 2020) in Qatar. A validated, closed-ended questionnaire was administered to explore participants’ views on audio-dentistry. The questionnaire explored the influence of variables related to dental problems depending on the specialty required, the years of experience of the responding dentist, and teletriage management decisions on overall satisfaction with audio-dentistry.

**Results:**

The response rate was 80.18%. Most participants expressed positive views of audio-dentistry in five domains (usefulness, interaction quality, ease of use and reliability, quality of care, satisfaction, and future use). However, approximately one-third of participants disagreed or strongly disagreed that their dental problem had improved following the call (35.3%) and viewed the lack of physical contact as a disadvantage (31.2%). Overall satisfaction was only influenced by telephone triage outcomes, with patients transferred for chairside management more likely to be satisfied (89.8%) than those managed remotely through self-care instructions and medications (80.4%) or instructions only (75.4%) (*p* = 0.011).

**Conclusions:**

Audio-dentistry effectively sustained oral health services during the COVID-19 pandemic while minimizing face-to-face visits, with patients largely expressing high satisfaction in areas such as usefulness, interaction quality, ease of use, reliability, and overall care. Satisfaction was primarily influenced by call outcomes and referrals or prescription decisions rather than caller demographics or dentist experience. However, some dissatisfaction arose when immediate improvement was not achieved, particularly in conditions such as pulpitis that are challenging to manage remotely.

## Introduction

Teleconsultation and telephone triage (teletriage) is the process of assessing calls from people with healthcare problems and managing the reported conditions through advice or referral to a more appropriate service.^1^ Teletriage has become widely accepted as a means of delivering healthcare,^2^ with a significant increase in its use during the COVID-19 pandemic.^3^ This is particularly true for dental services that were limited to emergency and urgent conditions during the pandemic, resulting in teledentistry, which uses telecommunications to provide consultations, treatment planning, and dental care remotely^4^ and is used to assess the need for emergency dental treatment while minimizing the risk of virus transmission.^3,4^ Teledentistry has ensured the continuity of emergency dental care during the pandemic while ensuring that both dental care teams and patients feel safe and experience less fear and anxiety.^5–7^

The Hamad Dental Centre (HDC), which is part of Hamad Medical Corporation (HMC) and the only public tertiary dental care provider in Qatar, established a hotline to maintain the continuity of essential oral and dental health services for dental emergencies during the COVID-19 pandemic.^8^ While the real-time (synchronous) mode of teledentistry involves both audioconferencing and videoconferencing,^9^ early in the pandemic, videoconferencing was not used and the full potential of teledentistry was not initially mobilized at the HDC because the associated major reorganization of dental care services was a challenge that required time. Therefore, audio-dentistry, a synchronous audioconferencing approach to delivering teledentistry services,^10^ formed a major part of this hotline.^8^

It is imperative to examine patients’ perceptions of the effectiveness of audio-dentistry and teletriage. Healthcare providers and patients often have different perceptions of the patient's health status and needs, level of satisfaction, opinions about the quality of communication during a medical visit, perceptions of the quality of care, and perceptions of stressors during healthcare delivery.^11^ Continuing to provide medical services despite this discrepancy between views may lead to a worse patient experience. Understanding patients’ perceptions could help address this mismatch and improve the patient experience. Furthermore, acceptance of telemedicine applications, including audio-dentistry and teletriage, is a prerequisite for obtaining potential benefits from the service,^12^ and such information can facilitate planning for future service provision.^13^ Patient satisfaction is an effective indicator of the success of healthcare services, and it influences clinical outcomes and patient retention rates.^14^ Therefore, it is important to investigate the effectiveness of audio-dentistry by conducting studies to examine service user satisfaction and perceptions of the usefulness of dental triage and the quality of communication and care.

Before the COVID-19 pandemic, limited evidence on patient perceptions of teledentistry services was published^15^ and research focused mainly on care providers^16–19^ who generally believed that teledentistry would improve dental practice. However, since the start of the COVID-19 pandemic, more studies on patient experiences with teledentistry have emerged.^13,20–25^ These studies generally reported positive patient experiences with teledentistry, but certain gaps in knowledge still exist, including underrepresented patient populations,^20^ small sample sizes, limited measures of effectiveness,^21^ and limited scope. None of the studies explored service users’ perceptions of the effectiveness of teletriage in dental emergencies, although emergency patients had the lowest satisfaction scores across various healthcare settings.^26^ Some studies investigated patients’ perceptions during new patient consultations and follow-up visits,^14–21^ while others explored patient attitudes during oral screening visits.^25^ One study explored patients’ perceptions of videoconferencing.^13^ No studies have examined the differences in overall caller satisfaction across different types of reported dental problems according to the specialty required, although studies have reported that dentists’ perceptions of the proposed benefits of teledentistry vary by dental specialty.^27^ Differences in patient satisfaction according to the dentist's years of clinical experience have not been explored, although reports suggest that dentist-related factors influence diagnostic accuracy^28,29^ and that emergency physician seniority has an impact on clinical efficiency and patient outcomes.^30^ Likewise, none of the previous studies evaluated the impact of teletriage outputs for dental emergencies and care decisions on patient satisfaction, although triage outputs in emergency departments significantly impact performance indicators associated with emergency services.^31^

To address these gaps, the aim of the research was to assess various aspects of service users’ perceptions (whether patients, their parents, or other caregivers) regarding the effectiveness of audio-dentistry teletriage for dental emergencies during the COVID-19 pandemic. The findings of the study can help improve the patient experience, maximize the potential benefit of the service, facilitate planning for future service provision, and influence clinical outcomes and patient retention rates.

## Methods

### Ethics, participants, setting, and procedures

Approval to conduct this retrospective cross-sectional study was granted by the Institutional Review Board (IRB) at HMC as a service evaluation project. A validated telephone questionnaire was administered to explore service users’ views on the usefulness, interaction quality, ease of use, reliability, quality of care, satisfaction, and likelihood of future use of audio-dentistry teletriage as measures of effectiveness.^32^ Differences in satisfaction levels across different dental problems according to the specialty required, the years of experience of the responding dentist, and teletriage management decisions were also assessed. The questionnaire was used to assess the audio-dentistry teletriage service provided by the HDC during the first wave of the COVID-19 pandemic in Qatar. For the study, a list of patients who used this service during the lockdown (*N* = 1,239) over a five-month period from March 29 to August 31, 2020 was retrieved. Demographic data and preferred contact information were retrieved from the data routinely collected for clinical audit purposes. All patients who called the hotline with self-reported dental emergencies were considered to have dental emergencies. A team of qualified dentists formally established the HDC hotline and developed algorithms based on international recommendations^33,34^ to triage the calls and make a tentative diagnosis, as well as guide the management of self-reported dental emergencies by providing remote care and/or referrals to a dental or hospital emergency facility. Of the 1,239 patients, only those with complete medical records and contact numbers were initially included in the study (865). These patients were contacted between October 1, 2020 and January 31, 2021. The authors were able to reach 439 patients, 87 of whom were unable or unwilling to participate. The remaining 352 patients agreed to participate and answered the questionnaire. Users who initially participated in the calls during teletriage, whether they were patients themselves or individuals acting on their behalf (i.e. a parent, another family member, or a caregiver), completed the questionnaire.

Five authors (SAA, AAA, RB, MDA, and SRA) attended two training sessions conducted by the principal author to agree on the content, structure, sequence of items, and response formats used in the questionnaire, in order to ensure its administration to the patient in a neutral manner and to ensure privacy and confidentiality. Study information was provided to the potential participants over the phone and verbal consent was obtained in the presence of a witness (one of the authors). Callers were excluded from the study if they were unable to communicate in one of the questionnaire languages (English or Arabic), if they refused to participate, or if they were under 18 years of age, and their parent did not consent to their participation. Questionnaire language was used according to the participants’ preferences. The interviewer read the items aloud to the participants over the phone. Confidentiality and privacy were ensured throughout the process as researchers performed their tasks in a dedicated office.

### Questionnaire

**Figure 1.**
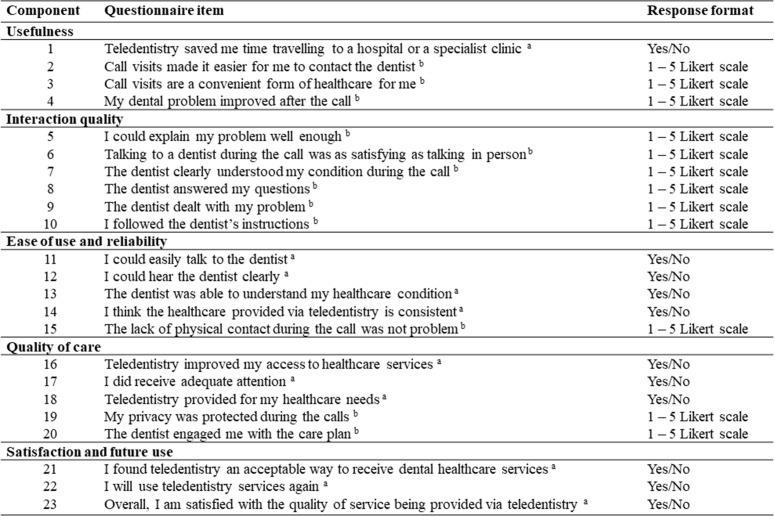
Service user questionnaire on the effectiveness of using the audio-dentistry form of teledentistry to teletriage dental emergencies.

The questionnaire was adopted from two validated and published questionnaires used to assess patients’ attitudes towards telemedicine.^12,35^ All questions included in the survey were closed-ended and had fixed responses (either yes/no or a five-point Likert scale: 5 = strongly agree, 4 = agree, 3 = neither agree nor disagree, 2 = disagree, and 1 = strongly disagree). Figure 1 shows the 23 items in the questionnaire that were divided into five domains/measures of effectiveness: usefulness (four questions), interaction quality (six questions), ease of use and reliability (five questions), quality of care (five questions), and satisfaction and future use (three questions).^32^

*Usefulness* refers to the caller's perception of the extent to which care provided by the audio-dentistry service is comparable to care provided during a conventional, in-person visit. Teletriage is useful when it functions appropriately and leads to positive clinical outcomes.^36^

*Interaction quality* measures the caller's interactions with the teledentist, including the quality of communication and the similarity of the experience to an in-person interaction.^35^

*Ease of use and reliability of teletriage* refers to the extent to which the service is easy for callers to learn and use without requiring excessive effort, as well as the ability of the service to facilitate the rapid completion of work and achieve its objectives effectively and satisfactorily.^37^

*Quality of care* refers to the overall quality of care received by callers and the benefit they perceive from following the teledentist's instructions and/or taking the prescribed medication.^35^

*Satisfaction and future use* are related to the overall satisfaction of callers with the teletriage service and the extent to which they would be willing to use the system in the future.^32^

### Validity of the questionnaire

Although the questionnaire was modified from two validated and published questionnaires,^12,35^ it was necessary to establish the translation validity and the face validity to ensure that the questions related to the audio-dentistry form of teledentistry and teletriage could be understood and interpreted appropriately by the intended study participants.^38^ To ensure the validity of the translation, the questionnaire was translated into Arabic using a professional translation service to ensure that the meaning did not change.^39^ It was also translated by the principal author, who is well versed in English and Arabic. These two versions were compared and discussed with the translators to develop a verified version. To ensure accuracy, back translation of the verified version into English was also performed.^39^ To assess face validity, four bilingual senior consultants from the fields of periodontics, orthodontics, and endodontics were approached to determine whether the content of the questionnaires (in English and Arabic) was relevant to the purpose of the study and appropriate for the setting in Qatar. The consultants evaluated whether the survey items successfully captured the intended topic, assessed item construction, and corrected errors such as leading, confusing, or double-barreled items as appropriate. The questionnaire was also assessed for clarity and accuracy, appropriateness for a household telephone survey, communication of the correct message, and consistency of style and formatting.^40^

### Statistical analysis

Descriptive statistics were used to characterize the sample. Overall differences in satisfaction across different types of dental problems were assessed using the Pearson chi-square test, according to the specialty required, years of clinical experience of the responding dentist, and teletriage management decisions. A *p* value < 0.05 (two-tailed) was statistically significant. Statistical analyses were performed using SPSS (Statistical Package for Social Sciences) software version 22.

## Results

### Sample characteristics

Of the 439 service users contacted, 352 agreed to participate, giving a response rate of 80.18%.

Details of the demographic characteristics of the sample are presented in Table 1. More females (61.1%) participated, the most common age group was ≤ 18 years of age (23.3%), and the majority were Qatari nationals (63.4%). Almost half of the respondents were employed, while the remaining participants were either unemployed, students, retired, self-employed, or preferred not to provide information. For patients aged ≤ 18 years, one of their parents participated in the study and answered the questionnaire. The majority of calls were made by the patients themselves, but 29% were made by a parent and 5.1% by a caregiver or first-degree relative. The mean duration of teletriage calls was 5.57+3.83 minutes, while the mean duration of survey calls was 12.83+8.82 minutes.

### Perceptions of service users

**Figure 2.**
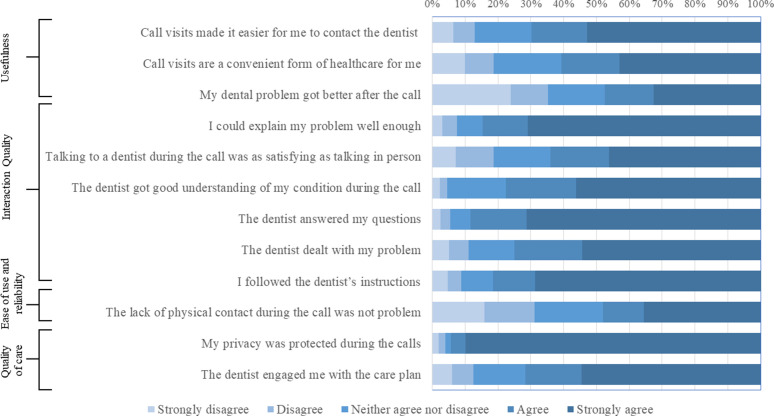
Service user questionnaire responses to five-point Likert scale questions.

**Figure 3.**
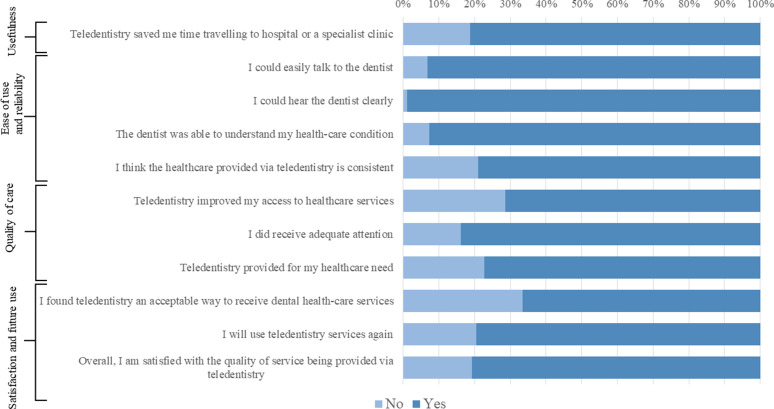
Service user questionnaire responses for yes/no questions.

Service users’ perceptions of the effectiveness of audio-dentistry were assessed by examining their responses to each domain of the questionnaire. Data on participants’ feedback on the effectiveness of teletriage are shown in Figures 2 and 3.

#### Usefulness

The majority of participants found the audio-dentistry visits useful and agreed/strongly agreed that these visits made it easier for them to contact the dentist (69.6%) and were a convenient form of healthcare (60.5%). The majority of participants (81%) affirmed that teledentistry saved time traveling to the hospital. However, 35.3% of the participants disagreed/strongly disagreed that their dental problem improved following the call. Of these participants, 32.2% were remotely diagnosed with pulpitis, 13.7% with broken or loose orthodontic appliances, and 11.3% with a broken or loose dental restoration/prosthesis. Only 56.5% of the group of participants who disagreed/strongly disagreed that their dental problem improved following the call agreed/strongly agreed that they had followed the dentist's instructions. However, more than half of the same group reported that they were satisfied overall with the quality of services provided (57.3%).

#### Interaction quality

Most participants were equally satisfied (64.9% agree/strongly agree) with talking to a dentist via audio-dentistry as in person. The majority of the participants agreed that the interaction quality during the calls was high on both ends of the call (the dentist's end and their own): the participants agreed/strongly agreed that they could adequately explain their problem (84.6%); that the dentist developed a good understanding of their condition (77.6%), answered their questions (88.3%), and addressed their problems (75%); and that they subsequently followed the dentist's instructions (81.6%).

#### Ease of use and reliability

Most participants expressed positive views about the ease and reliability of using the telephone to report their dental emergency and receive the teletriage service. They could talk to the dentist easily (93.2%) and hear them clearly (98.9%). Participants also reported that the dentist was able to understand their healthcare condition (92.6%) and that the healthcare provided via teledentistry was consistent (79%). However, the lack of physical contact during the call was viewed as a disadvantage by approximately one-third of the participants (31.2%).

#### Quality of care

The feedback on the quality of care provided via the audio-dentistry teletriage service was positive. Most respondents affirmed that audio-dentistry improved their access to healthcare (71.3%) and met their healthcare needs (77.3%). Most participants (83.8%) reported that they received adequate attention, agreed/strongly agreed (71%) with statements indicating that the dentist engaged them with the care plan, and that their privacy was protected during the calls (94.3%).

#### Satisfaction and future use

Responses to the virtues of audio-dentistry teletriage were positive, with two-thirds of the participants viewed teledentistry as an acceptable way to receive dental healthcare services (66.5%), and 79.5% stated that they would use teledentistry services in the future. Overall, 80.7% of the participants were satisfied with the quality of audio-dentistry teletriage of emergency dental service during the COVID-19 pandemic.

### Factors influencing the overall satisfaction of teletriage service users

A significant majority of the study participants (80.7%) expressed overall satisfaction with the teledentistry service. We examined the factors that influenced service users’ overall satisfaction with audio-dentistry services. Table 2 shows the differences in overall satisfaction across several variables. Overall, participants’ satisfaction did not vary by sex, age group, the type of dental problem according to the specialty required or the dentist's years of experience. However, overall satisfaction varied depending on the decision to use audio-dentistry teletriage, with patients who were transferred to in-person management being more satisfied (89.8%) than those managed remotely through the dissemination of instructions and medications (80.4%) or through the dissemination of instructions only (75.4%) (*p* = 0.011).

## Discussion

Audio-dentistry was commonly used during the first wave of the COVID-19 pandemic,^41^ and it achieved its goals by maintaining the continuity of oral health services while minimizing in-person visits.^8^ Understanding service users’ perceptions of teledentistry is not only important for measuring the success of the service and its providers, but also for planning similar situations in the future. As far as is known at the time of manuscript submission, this study is the first to evaluate patients’ perceptions of the effectiveness of audio-dentistry teletriage in self-reported dental emergencies. It was found that most participants were satisfied with the use of audio-dentistry across the five domains explored: usefulness, interaction quality, ease of use and reliability, quality of care, satisfaction, and future use. Caller demographics, type of dental problem, and the dentist's years of clinical experience did not influence users’ overall satisfaction with the service. Overall satisfaction was influenced by the outcome of the call and whether the patient was referred for in-person care or prescribed medication. This may be because patients or their caregivers contacted the service with a sense of urgency and were likely expecting an in-person referral. During the COVID-19 pandemic, teletriage decisions were heavily influenced by patient-reported pain severity,^42^ such that patients who felt their condition as serious but did not report severe pain could have expected to be considered urgent and referred for in-person care. This unmet expectation could have impacted their overall satisfaction with the service.

Despite overall satisfaction with the service provided, there was some dissatisfaction in certain aspects. For example, nearly one-third of the participants felt that their dental problems did not improve following the call. These perceptions may be partly attributed to the fact that less than half of the respondents followed the dentist's instructions. Additionally, 32.2% were remotely diagnosed with painful pulpitis. During the pandemic, not only was it difficult to diagnose patients with pulpitis remotely,^10^ but they were also not immediately referred for in-person management, but only after trying to control pain severity with medication. Similarly, patients with problems associated with orthodontic appliances or dental restorations were referred for in-person management only if the problem posed a risk to the caller's overall health or impacted on quality of life. These findings may suggest the need for improvement in remote pain control strategies and/or medications, as well as the development of affordable dental emergency home kits that can help patients continue their daily activities while waiting for their chairside visits.

Although the participants’ perceptions of teledentistry and their satisfaction with the audio-dentistry teletriage service were generally positive, the percentage of participants reporting satisfaction was lower than that reported by previous studies on the use of audio-dentistry for consultation and follow-up visits, especially with respect to the domains of usefulness, ease of use, and interaction quality. For example, when asked whether teledentistry saved time traveling to the hospital, only 81% of the study participants answered “yes”, while 100% of participants in a UK study agreed that virtual clinic saved them time related to travel, work, or other commitments.^21^ Similarly, when asked whether talking to a dentist during the call was as satisfying as talking in person, only 64% of callers agreed/strongly agreed, while 100% of respondents in the UK study agreed that they could talk to the dentist both via teledentistry and in person.^21^ These differences may be due to the urgency of the calls received by the HDC team, as the study participants called to report dental emergencies rather than to schedule a follow-up visit with a familiar dentist. This difference is not surprising since satisfaction ratings for emergency patients are often lowest among a variety of healthcare settings.^26^ Additionally, given the larger geographical size of the UK compared to Qatar, distances to central facilities are significantly greater in remote areas of the UK. Furthermore, telemedicine services were new to the public in Qatar, having been introduced during the pandemic.^8^ This was different from the UK, where many patients in the previous study had previously used telehealth services.^21^ However, the sample size of the present study was significantly larger than those reported in previous studies, which improves the reliability of the responses. The callers reported overall positive satisfaction, which could be attributed to their satisfaction with the quality of care despite the lack of improvement in their symptoms. This finding confirms previous reports that the service attitude of medical staff is the most important factor in patient satisfaction.^43^

This retrospective study had its limitations. The telephone-based questionnaire was not anonymous, potentially influencing patients’ willingness to provide negative feedback. Additionally, the survey was conducted six months after the audio-dentistry call, introducing possible recall bias.^44^ Future electronic-based prospective surveys could improve reliability. Despite these limitations, the survey achieved a response rate of 80.18%, representing a good cross-section of the study population.^45^

The primary public health implication of the findings in this paper is that there are significant advantages to extending the use of audioconferencing, particularly in situations where callers are not tech-savvy or are uncomfortable with new methods of interaction. Additionally, videoconferencing can be challenging due to network service issues that impact the quality of consultations and therefore patient satisfaction and acceptance of teledentistry.^13,21^ Moreover, access to videoconferencing can be difficult for elderly individuals, people from socioeconomically disadvantaged backgrounds, or those with certain physical or learning limitations.^21^ Therefore, it is worthwhile to investigate the effectiveness of audioconferencing in teletriage.

Future studies are needed to assess outcomes by comparing audio-dentistry and videoconferencing visits or comparing synchronous and asynchronous forms of teledentistry and their applications to develop better emergency teletriage services in dentistry. The present study highlighted the need for improvement in remote pain control strategies. Therefore, further research is needed to help patients who are deprived of robust and easy access to chairside dental care to relieve their symptoms. Research to develop affordable dental emergency home kits is also essential to help patients continue their daily activities while waiting for a chairside care visit.

## Conclusions

Audio-dentistry has proven to be a vital tool in maintaining oral health services during the COVID-19 pandemic, significantly reducing the need for in-person visits. This pioneering study found high patient satisfaction in areas such as usefulness, interaction quality, ease of use, reliability, and quality of care, with satisfaction driven by outcomes rather than demographic characteristics. However, challenges remain, particularly in remotely managing conditions such as pulpitis.

The way forward is clear: expanding audioconferencing could be transformative, especially for those who are less comfortable with video interactions, have limited internet access, or are not tech-savvy. Future research should explore the full potential of teledentistry, comparing audio and video approaches, innovating in remote pain management, and developing affordable emergency dental kits. These initiatives are crucial for enhancing patient care and bridging the gap while awaiting in-person visits.

### Ethics approval

The IRB at HMC considered this analysis as an audit or service/therapy evaluation project since the data were collected for service audits and as an integral part of service evaluation purposes. Therefore, the project was granted an exemption from requiring ethics approval.

### Informed consent statement

Informed consent was obtained from all subjects involved in the study. All participants agreed to participate in the questionnaire over the phone.

### Competing interests

The authors have no competing interests to declare.

## Figures and Tables

**Table 1.** Demographic characteristics of the sample (*n* = 352).

**Characteristic**	*N* (%)

**Sex**	

**Female**	215 (61.1)

**Male**	137 (38.9)

Age (years)*	

** ≤ 18**	82 (23.3)

**19–29**	75 (21.3)

**30–39**	74 (21.0)

**40–49**	51 (14.5)

**50–59**	40 (11.4)

**60–69**	23 (6.5)

** ≥ 70**	7 (2.0)

**Nationality**	

**Qatari**	223 (63.4)

**Arab (other than Qatari)**	96 (27.3)

**Asian**	26 (7.4)

**European/North American**	7 (2.0)

**Employment status**	

**Employed**	169 (48.0)

**Unemployed**	92 (26.1)

**Student**	49 (13.9)

**Retired**	26 (7.4)

**Prefer not to say**	12 (3.4)

**Self-employed**	4 (1.1)

**Respondent/patient relationship**	

**Caller is the patient himself or herself**	232 (65.9)

**Mother**	74 (21.0)

**Father**	28 (8.0)

Other**	18 (5.1)

**Duration of calls (M ± SD)**	

**Teletriage calls**	5.57 ± 3.83

**Telephone questionnaire calls**	12.83 ± 8.82


*10-year age groups employed according to the WHO standards; **caregiver or first-degree relative; M ± SD = mean ± standard deviation.

**Table 2.** Differences in overall satisfaction depending on patient sex, age group, different types of dental problems according to the specialty required, the dentist's years of clinical experience, and teletriage management decisions.

	Total^a^	Overall, I am satisfied^b^		

Characteristic	*N* (%)	No	Yes	*p* ^c^

Whole sample	352 (100)	68 (19.3)	284 (80.7)	

Sex				0.897

Female	215 (61.1)	42 (19.5)	173 (80.5)	

Male	137 (38.9)	26 (19)	111 (81)	

Age (years)				0.795

≤ 18	82 (23.3)	19 (23.2)	63 (76.8)	

19–29	75 (21.3)	15 (20)	60 (80)	

30–39	74 (21.0)	14 (18.9)	60 (81.1)	

40–49	51 (14.5)	6 (11.8)	45 (88.2)	

50–59	40 (11.4)	9 (22.5)	31 (77.5)	

60–69	23 (6.5)	4 (17.4)	19 (82.6)	

≥ 70	7 (2.0)	1 (14.3)	6 (85.7)	

Teletriage management decision (*N* = 351)				0.011

Referred for face-to-face care	108 (30.8)	11 (10.2)	97 (89.8)	

Remote management with instructions+medications	56 (16.0)	11 (19.6)	45 (80.4)	

Remote management with instructions	187 (53.3)	46 (24.6)	141 (75.4)	

Type of dental problem according to the specialty required (*N* = 351)				0.242

Prosthodontic	37 (10.5)	11 (29.7)	26 (70.3)	

Pedodontics	25 (7.1)	7 (28)	18 (72)	

Endodontic	73 (20.8)	17 (23.3)	56 (76.7)	

General dentistry	25 (7.1)	5 (20)	20 (80)	

Orthodontic	101 (28.8)	17 (16.8)	84 (83.2)	

Periodontic	20 (5.7)	3 (15)	17 (85)	

Oral surgery	70 (19.9)	8 (11.4)	62 (88.6)	

Dentist's years of clinical experience (*N* = 351)				0.265

≥ 20	51 (14.5)	7 (13.7)	44 (86.3)	

11–19	182 (51.8)	33 (18.1)	149 (81.9)	

1–10	118 (33.6)	28 (23.7)	90 (76.3)	


Cells represent frequency and rows represent percentages unless otherwise indicated; ^a^ percentages in this column are column percentages; ^b^Overall, I am satisfied with the quality of the services provided via teledentistry; ^c^The *p* value was based on chi-square (with 95% CI).

## References

[bib1] South Wiltshire Out of Hours Project (SWOOP) Group (1997). Nurse telephone triage in out of hours primary care: A pilot study. BMJ.

[bib2] Allemann Iseli M, Kunz R, Blozik E (2014). Instruments to assess patient satisfaction after teleconsultation and triage: A systematic review. Patient Prefer Adherence.

[bib3] Kumar U, Gupta A, Goyal A, Gauba K (2021). Impact of COVID-19 pandemic on characteristics of dental emergencies and treatment services at a tertiary care centre. Saudi Dent J.

[bib4] Meng L, Hua F, Bian Z (2020). Coronavirus disease 2019 (COVID-19): Emerging and future challenges for dental and oral medicine. J Dent Res.

[bib5] Goriuc A, Sandu D, Tatarciuc M, Luchian I (2022). The impact of the COVID-19 pandemic on dentistry and dental education: A narrative review. Int J Environ Res Public Health.

[bib6] Estai M, Kanagasingam Y, Tennant M, Bunt S (2018). A systematic review of the research evidence for the benefits of teledentistry. J Telemed Telecare.

[bib7] Barsom EZ, Feenstra TM, Bemelman WA, Bonjer JH, Schijven MP (2020). Coping with COVID-19: Scaling up virtual care to standard practice. Nat Med.

[bib8] Ali SA, Al-Qahtani AMA, Al Banai SR, Albaker FJ, Almarri AE, Al-Haithami K (2022). Role of newly introduced teledentistry service in the management of dental emergencies during COVID-19 pandemic in Qatar: A cross-sectional analysis. Telemed J E Health.

[bib9] AlShaya MS, Assery MK, Pani SC (2020). Reliability of mobile phone teledentistry in dental diagnosis and treatment planning in mixed dentition. J Telemed Telecare.

[bib10] Ali SA, El Ansar (2022). W. Is tele-diagnosis of dental conditions reliable during COVID-19 pandemic? Agreement between tentative diagnosis via synchronous audioconferencing and definitive clinical diagnosis. J Dent.

[bib11] Natafgi N, Ladeji O, Blackwell S, Hong YD, Graham G, Cort M (2022). Similar values, different expectations: How do patients and providers view ‘health’ and perceive the healthcare experience?. Health Expect.

[bib12] Bakken S, Grullon-Figueroa L, Izquierdo R, Lee NJ, Morin P, Palmas W (2006). Development, validation, and use of English and Spanish versions of the telemedicine satisfaction and usefulness questionnaire. J Am Med Inform Assoc.

[bib13] Menhadji P, Patel R, Asimakopoulou K, Quinn B, Khoshkhounejad G, Pasha P (2021). Patients’ and dentists’ perceptions of tele-dentistry at the time of COVID-19: A questionnaire-based study. J Dent.

[bib14] Prakash B (2010). Patient satisfaction. J Cutan Aesthet Surg.

[bib15] Daniel SJ, Wu L, Kumar S (2013). Teledentistry: A systematic review of clinical outcomes, utilization and costs. J Dent Hyg.

[bib16] Estai M, Kruger E, Tennant M (2016). Perceptions of Australian dental practitioners about using telemedicine in dental practice. Br Dent J.

[bib17] Mandall NA, Qureshi U, Harvey L (2005). Teledentistry for screening new patient orthodontic referrals. Part 2: GDP perception of the referral system. Br Dent J.

[bib18] Palmer NG, Yacyshyn JR, Northcott HC, Nebbe B, Major PW (2005). Perceptions and attitudes of Canadian orthodontists regarding digital and electronic technology. Am J Orthod Dentofacial Orthop.

[bib19] Flores-Mir C, Palmer NG, Northcott HC, Khurshed F, Major PW (2006). Perceptions and attitudes of Canadian dentists toward digital and electronic technologies. J Can Dent Assoc.

[bib20] Omezli MM, Torul D, Yilmaz EB (2022). Is teledentistry a feasible alternative for people who need special care?. Disaster Med Public Health Prep.

[bib21] Rahman N, Nathwani S, Kandiah T (2020). Teledentistry from a patient perspective during the coronavirus pandemic. Br Dent J.

[bib22] Heimes D, Luhrenberg P, Langguth N, Kaya S, Obst C, Kämmerer PW (2022). Can teledentistry replace conventional clinical follow-up care for minor dental surgery? A prospective randomized clinical trial. Int J Environ Res Public Health.

[bib23] Amtha R, Gunardi I, Astoeti TE, Roeslan MO (2021). Satisfaction level of the oral medicine patients using teledentistry during the COVID-19 pandemic: A factor analysis. J Int Soc Prev Community Dent.

[bib24] Torul D, Kahveci K, Kahveci C (2021). Is tele-dentistry an effective approach for patient follow-up in maxillofacial surgery. J Maxillofac Oral Surg.

[bib25] Aboalshamat KT, Althagafi TK, Alsaeedi SA, Alhumaidi SN, Alemam AA (2022). Accuracy and perceptions of teledentistry in KSA during the COVID-19 pandemic: A single-centre randomised controlled trial. J Taibah Univ Med Sci.

[bib26] Abass G, Asery A, Al Badr A, AlMaghlouth A, AlOtaiby S, Heena H (2021). Patient satisfaction with the emergency department services at an academic teaching hospital. J Family Med Prim Care.

[bib27] Al-Khalifa KS, AlSheikh R (2020). Teledentistry awareness among dental professionals in Saudi Arabia. PLoS One.

[bib28] Radic J, Patcas R, Stadlinger B, Wiedemeier D, Rücker M, Giacomelli-Hiestand B (2018). Do we need CBCTs for sufficient diagnostics?-dentist-related factors. Int J Implant Dent.

[bib29] Hellén-Halme K, Petersson GH Influence of education level and experience on detection of approximal caries in digital dental radiographs: An in vitro study. Swed Dent J.

[bib30] Li CJ, Syue YJ, Tsai TC, Wu KH, Lee CH, Lin YR (2016). The impact of emergency physician seniority on clinical efficiency, emergency department resource use, patient outcomes, and disposition accuracy. Medicine (Baltimore).

[bib31] Casalino E, Choquet C, Bernard J, Debit A, Doumenc B, Berthoumieu A (2013). Predictive variables of an emergency department quality and performance indicator: A 1-year prospective, observational, cohort study evaluating hospital and emergency census variables and emergency department time interval measurements. Emerg Med J.

[bib32] Parmanto B, Lewis AN Jr, Graham KM, Bertolet MH (2016). Development of the Telehealth Usability Questionnaire (TUQ). Int J Telerehabil.

[bib33] Centers for Disease Control and Prevention. Guidance for dental settings: Interim infection prevention and control guidance for dental settings during the COVID-19 response 2020 Available from: https://www.cdc.gov/coronavirus/2019-ncov/hcp/dental-settings.html

[bib34] World Health Organization Consideration for the provision of essential oral health services in the context of COVID-19: Interim guidance. 2020 Available from: https://www.who.int/publications/i/item/who-2019-nCoV-oral-health-2020

[bib35] Yip MP, Chang AM, Chan J, MacKenzie AE (2003). Development of the Telemedicine Satisfaction Questionnaire to evaluate patient satisfaction with telemedicine: A preliminary study. J Telemed Telecare.

[bib36] Ekeland AG, Bowes A, Flottorp S (2010). Effectiveness of telemedicine: A systematic review of reviews. Int J Med Inform.

[bib37] ISO Ergonomic requirements for office work with visual display terminals (VDTs) Part 1: General introduction Int Org Stand 1992 Available from: https://www.iso.org/iso/iso_catalogue/catalogue_ics/catalogue

[bib38] Tsang S, Royse CF, Terkawi AS (2017). Guidelines for developing, translating, and validating a questionnaire in perioperative and pain medicine. Saudi J Anaesth.

[bib39] Singh R, Agarwal TM, Al-Thani H, Al Maslamani Y, El-Menyar A (2018). Validation of a survey questionnaire on organ donation: An Arabic world scenario. J Transplant.

[bib40] Mitchell RC, Carson RT (1989). Using surveys to value public goods: The contingent valuation method.

[bib41] Weintraub JA, Quinonez RB, Smith AJT, Ciarrocca K, Fouad AF, Shazib MA (2020). Responding to a pandemic: Development of the Carolina Dentistry Virtual Oral Health Care Helpline. J Am Dent Assoc.

[bib42] Ali SA, El Ansari W (2022). Patient-reported orofacial-dental pain severity and tele-triage decisions during COVID-19 pandemic: Does the severity of pain drive tele-triage decisions?. BMC Oral Health.

[bib43] Fang J, Liu L, Fang P (2019). What is the most important factor affecting patient satisfaction – a study based on gamma coefficient. Patient Prefer Adherence.

[bib44] Jensen J, Hansen CF, Brodersen J, Comins JD, Krogsgaard MR (2021). Are PROMs used adequately in sports research? An analysis of 54 randomized controlled trials with PROMs as endpoint. Scand J Med Sci Sports.

[bib45] Fincham JE (2008). Response rates and responsiveness for surveys, standards, and the Journal. Am J Pharm Educ.

